# Long-term outcomes of spinal fusion in adolescent idiopathic scoliosis: a literature review

**DOI:** 10.1186/s40001-024-02052-7

**Published:** 2024-11-05

**Authors:** Miguel Pishnamaz, Filippo Migliorini, Christian Blume, Philipp Kobbe, Per Trobisch, Heide Delbrück, Frank Hildebrand, Christian Herren

**Affiliations:** 1 Department of Orthopaedics, Trauma and Reconstructive Surgery, RWTH Aachen University, Pauwelsstraße 30, 52074 Aachen, Germany; 2 Department of Orthopaedic and Trauma Surgery, Academic Hospital of Bolzano (SABES-ASDAA), 39100 Bolzano, Italy; 3 Department of Life Sciences, Health, and Health Professions, Link Campus University, 00165 Rome, Italy; 4 Department of Neurosurgery, RWTH Aachen University, 52074 Aachen, Germany; 5 Department of Trauma and Reconstructive Surgery, BG Klinikum Bergmannstrost, 06112 Halle, Germany; 6 Department of Trauma, Hand and Reconstructive Surgery, Martin-Luther-University Halle-Wittenberg, Ernst-Grube-Strasse 40, 06120 Halle (Saale), Germany; 7 Department of Spine Surgery, Eifelklinik St. Brigida, 52152 Simmerath, Germany

**Keywords:** Adolescent idiopathic scoliosis, AIS, Long-term outcome, Scoliosis, Posterior fusion

## Abstract

Adolescent idiopathic scoliosis (AIS) is the most common form of spinal deformity in the younger population. The surgical management for these patients improved constantly over the last year and might not be comparable to modern treatment strategies. However, under this aspect the present investigation updates and discusses current evidence regarding the long-term outcome of the surgical management of AIS. All the clinical studies which evaluated the long-term outcomes of spinal fusion were considered. Level of evidence, clinical and radiological data, results of health-related questionnaires and surgery-associated complications during long-term follow-up, e.g., proximal and distal junctional kyphosis (PJK/DJK), and adjacent segment degeneration (ASD), are presented. Data concerning the following patient-reported outcomes measures were collected: Oswestry Disability Index (ODI), Scoliosis Research Society (SRS) Outcome Questionnaire, visual analogue scale (VAS), and short form-12 and 36 (SF-12/SF-36). Overall, data from 1115 patients were included. Of them, 324 underwent anterior and 791 posterior spinal fusion. One study focuses on a combined anterior/posterior fusions. The mean follow-up was 22.6 years (posterior fusion: 24.6 years, anterior fusion: 18.31 years). Seven studies focus on the thoracic segments, while 12 focus on the lumbar spine. Data on imaging was reported in 13 studies and those on PROMs in 15 investigations. In conclusion, there is low quality and paucity of long-term data on AIS. However, the long-term results of the implicated studies on AIS patients in this review appear to be satisfactory, although there are limitations in the outcome compared to healthy comparison cohorts. Adjacent degenerations appear to be the most common mechanical complication after long-segment fusions, despite their influence on the outcome remains unclear. With regard to pregnancies, there are slightly increased cesarean section rates, which could be explained by deviations in the sagittal profile.

## Introduction

Adolescent idiopathic scoliosis (AIS) is the most common cause of spinal deformity in children and the most common form of scoliosis in general [1, 2]. The treatment of AIS is complex, time-consuming and expensive. The therapeutic options include conservative therapy with or without bracing, dynamic fixations and, if necessary, extensive spinal fusions [3]. Close monitoring during the growth phase is necessary to supervise the success of the therapy and to identify progressive malformations at an early stage [4, 5]. While physiotherapy and orthoses remain the core elements of conservative therapy, the progressive development of modern spinal fixation systems has led to an improvement in surgical therapy options.

Harrington rods were among the first-generation systems for the surgical treatment of AIS, but their use was fraught with complications, which led to unsatisfactory results given the one-dimensional correction of the deformity in the coronary plane [6–8]. Nowadays, posterior pedicle screw-based internal fixation systems offer the possibility of three-dimensional correction. By correct usage of these systems and by taking into account the sagittal profile of the patients during surgical planning, the typical iatrogenic flat back deformity can be avoided in many cases [9, 10].

Furthermore, growth-guiding systems, such as vertebral body tethering systems (VBT), are on the rise for the management of younger adolescent idiopathic scoliosis (AIS) patients with expected residual growth. These techniques aim to maintain mobility and avoid fusion if possible. In addition, even though the optimal criteria for the best candidates have yet to be defined, the use of growth guiding systems means that fusion procedures are increasingly only required in patients where the phase of residual growth has been missed or where the deformity of the spine cannot be adequately addressed using dynamic methods [11–13].

Regardless of the surgical approach, therapy aims to correct the Cobb angle, restore the balance of the trunk in the sagittal and coronal planes, symmetrise the waist and shoulder position, and correct the rotational misalignment while avoiding complications in the long-term follow-up [9, 14]. In this context, the length of the instrumentation should always be as long as necessary and as short as possible [14]. Nevertheless, a change in biomechanics by lengthening the lever arm and reducing the motion segments remains unavoidable in fusion surgeries. In this context, proximal or distal junctional kyphosis (PJK and DJK) or adjacent segment degeneration (ASD) are known complications [15–18].

Considering that the constant improvement of surgical procedures in AIS has occurred in recent years, we aimed to find out whether long-term results are already available that can be used to treat AIS today. Four specific questions regarding outcome were addressed: (1) what is the benefit of surgery for AIS in correcting the main curve at a minimum follow-up of 15 years? (2) What is the benefit of surgery for AIS based on standard health-related questionnaires at a minimum follow-up of 15 years? (3) What are the main mechanical complications following surgical intervention for AIS? and (4) What limitations can be expected in a future pregnancy after surgical treatment of AIS?

## Methods

All the clinical investigations that evaluated the surgical management of AIS were retrieved. Only studies with a minimum of 15 years of follow-up were eligible. A Review of Clinical Evidence of the English and German literature (PubMed and Cochrane Library; 2007–2023) was performed using the following search terms: “idiopathic scoliosis” and “long-term”. The bibliographies of the retrieved articles were also searched by hand to identify potentially relevant articles. The following string was used to search PubMed: ((("2007/01/01"[Date – Publication]: "3000"[Date – Publication])) AND (idiopathic scoliosis)) AND (long-term outcome). Titles and abstracts were reviewed. Studies which reported data on anterior and posterior fusion procedures were included. Studies that reported data on dynamic fusions (e.g., anterior vertebral body tethering) were excluded. Studies solely concentrating on Harrington rods were excluded. However, studies that evaluated Harrington rods combined with other surgical procedures were eligible. Case reports or series that included less than five patients were not considered. Studies which focus on conservative management were excluded. Studies with levels I–III, according to the Oxford Centre of Evidence-Based Medicine [19], were considered.

All articles were reviewed by three authors (CH, MP, PT, and FM) and discussed with others (FH, HD, PK, CB). A decision was made regarding inclusion and assessment of the level of evidence. If there was any disagreement among authors regarding the inclusion of an article, the majority decision was reached. The level of evidence was subsequently determined by the consensus of the authors involved, taking into account the level of evidence.

## Results

**Fig. 1:**
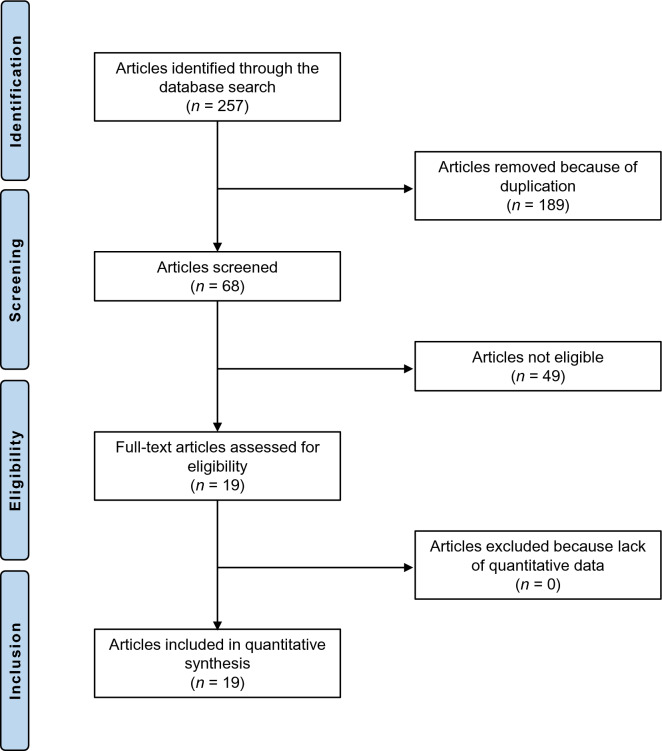
Flowchart showing publications included

Of the 257 articles identified, 19 publications were included (Fig. 1).

Overall, data from 1115 patients were retrieved. Of them, 324 underwent anterior and 791 posterior spinal fusion. One study focuses on a combined anterior/posterior fusion. The mean follow-up was 22.6 years (posterior fusion: 24.6 years, anterior fusion: 18.31 years). Seven studies focus on the thoracic segments, and 12 focus on the lumbar spine. Data on imaging was reported in 13 studies and those on PROMs in 15 investigations. Generalities and characteristics of the included studies are shown in Table 1.

**Table 1:** Generalities and characteristics of the included studies

Author	Type of treatment	Spine region	Number of patients	Mean follow-up time (years)	Outcome parameter	Key message	Evidence level
Akazawa T. et al	Posterior fusion (screw/rod system)	Thoracolumbar spine	256	31.5	Health-related Quality of Life (HrQoL)	Spinal surgery in AIS had no demonstrable adverse effects on pain or mental health in these middle-aged AIS patients 21–41	3b
Darnis, A. et al	Posterior fusion (screw/rod system)	Thoracic spine	109	17.6	Radiographic parameters, HrQoL	The reduction in the coronal plane is stable over the time; the HRQoL is quite as good as the general population	3b
Hamzaoglu, A. et al	Posterior fusion (screw/rod system)	Thoracic spine	43	18.7	Radiographic parameters, HrQoL	In this group of patients, the uninstrumented lumbar curve spontaneously corrected and the correction was maintained after 18 years following thoracic fusion	3b
Lohnstein, J.E	Posterior fusion (49 pediclescrews/hooks)	Thoracic spine	40	32,7	Radiographic parameters, HrQoL	The lumbar curve in selective thoracic fusions is unchanged, with patients functioning well and mild radiographic changes in the lumbar spine	3b
Kino, K. et al	Posterior fusion (CD-technique)	Thoracolumbar spine	87	27.5	Radiographic parameters, HrQoL	Although HrQoL scores (SF-36) in surgically treated women for AIS were lower than those of the healthy control group, the effects of posterior spinal fusion using CDI on women's social life and marital and reproductive statuses were minimal	3b
Akazawa, T. et al	Posterior fusion (screw/rod system)	Thoracolumbar spine	56 (non-idiopathic) vs. 80 (idiopathic)	21.0	HrQoL	The patients with non-IS and IS had similar health-related quality of life and low back pain	3b
Swany, L. et al	Posterior fusion (screw/rod system)	Thoracolumbar/lumbar Spine	60 (32 (operative) vs. 28 (conservative))	43.0	Pregnancy outcomes	In this long-term cohort of 60 US scoliosis patients, childhood operative fusion treatment was associated with a statistically significant increased incidence of C-section compared to the state incidence at both the patient level and the delivery level	3b
Lavelle, W.F. et al	Posterior fusion (screw/rod system)	Thoracolumbar spine	22	26.0	HrQoL	Most patients who underwent multi-segment spinal fixation appeared to do well long-term, with minimal back pain. Lowest instrumented segment did not appear to be associated with increased back pain	3b
Takayama, K. et al	Posterior fusion (screw/rod system)	Thoracolumbar/lumbar spine	32 (18 with AIS)	21.1	Radiograpic parameters, HrQoL, Low back pain	Positive sagittal balance at the latest follow-up was a factor significantly contributing to LBP following scoliosis surgery	3b
Yamada, K. et al	Posterior fusion (screw/rod system)	Thoracic spine	25	18.2	Radiographic parameters, HrQoL	The upper instrumented vertebra translation should be considered an important factor that influences postoperative results	3b
Takayama, K. et al	Posterior fusion (screw/rod system)	Thoracolumbar/lumbar spine	32 (18 with AIS)	21.1	HrQoL; Working status, marital status, and childbearing, clinical and radiologic evaluation	Larger preoperative Cobb angle and positive sagittal balance at the most recent follow-up were related to poor outcome in QOL as assessed by the SRS-22	3b
Akazawa, T. et al	Posterior fusion (screw/rod system)	Thoracolumbar/lumbar spine	42	21.0	HrQoL	Those scoliosis patients who underwent spinal fusion during adolescence had good HRQOL scores in midlife	3b
Larson, A.N	Posterior fusion	Thoracic spine	19	20.0	Radiographic parameters (lumbar curve)	Spinal balance and correction of the lumbar curve remain stable over time in selective thoracic fusion. Those with selective fusions have outcome measures comparable with those with long fusions	3b
Böhm, H. et al	Simultaneous anterior/posterior fusion	Thoracic spine	161	18.0	Radiographic parameters	Thoracoscopically assisted anterior release at the same time as a posterior standard scoliosis procedure is a justified and effective tool, yielding better results and maintaining them	3b
Delfino, R. et al	Anterior fusion (single rod system)	Thoracolumbar spine	42	17.3	Radiographic parameters, HrQoL	In the long term, selective anterior thoracolumbar instrumentation with a single solid rod in AIS maintained good corrections on the three planes with no major complications or infections	3a
Kelly, D.M. et al	Anterior fusion	Thoracolumbar spine	31	16.97	Radiographic parameters, HrQoL	The anterior approach in the treatment of thoracolumbar and lumbar curves in AIS offers good long-term functional outcomes for patients	3b
Sudo, H. et al	Anterior fusion	Thoracic spine	25	15.2	Radiographic parameters, HrQoL	Percent-predicted values of forced vital capacity and forced expiratory volume in 1 s were decreased after selective anterior fusion; however, no patient had complaints related to pulmonary function	3b
Sudo, H. et al	Anterior fusion (dual rod system)	Thoracolumbar/lumbar spine	30	21.4	Radiographic parameters, HrQoL	Short fusion strategy can be considered as an alternative to the conventional strategy in patients with thoracolumbar/lumbar AIS curves undergoing anterior spinal fusion with dual-rod instrumentation	3b
Rioullon, G. et al	Anterior fusion (screw-plate fixation)	Thoracolumbar/lumbar spine	35	21.0	Radiographic parameters, HrQoL	Anterior spinal fusion produces good long-term functional outcomes in AIS. Correction is both satisfactory and sustained	3b

## Discussion

The surgical treatment of idiopathic scoliosis is complex and requires individual therapy. Therefore, a homogenisation of the patient population would be desirable for evaluating therapies. Unfortunately, such a data set does not exist nowadays; hence, this review aims to summarise the long-term results of surgically treated scoliosis.

### Surgical approach

An important aspect when choosing the surgical approach is the possibility of correction. Nowadays, severe AIS is most commonly treated surgically through posterior fusion, given the greater possibility of deformity correction than anterior procedures [20, 21]. In this context, one major step was made in 1984 by Cotrel and Debousset (CD), who recommended a segmental spinal correction technique [22]. This technique was the first aimed at 3-D deformity correction and still represents a core element within the posterior instrumentations of spinal deformities [22, 23]. The CD technique provides a far better opportunity for three-dimensional scoliosis correction, and iatrogenic flat back is less likely to occur in the postoperative follow-up. Nowadays, other reduction techniques, such as dual differential, are also applied, and a combination of different techniques can be used in complex cases to achieve the best surgical result. Modern and potent screw-rod systems further reinforce the power of all those correction techniques. However, anterior procedures also offer the possibility of sufficient correction depending on the respective curve type.

Despite this, correction of a thoracic hyperkyphosis or rigid rib hump remains a problem in the posterior correction of idiopathic scoliosis in some cases. Recent literature suggests an additional anterior release to fill this gap. Böhm et al. described good radiographical results with good maintenance for up to 22 years. They reported a correction of 75% of the thoracic curve and an indirect reduction of the rib hump of 2.2 cm [9]. Nevertheless, some spine surgeons also trust standalone anterior instrumentation of thoracolumbar curves in adolescents [24, 25]. The advantages of the anterior approach include the possibility of an anterior release and, in certain circumstances, shorter fusions than the posterior approach [26–30].

 Delfino et al reported long term outcomes of over 17 years after selective anterior thoracolumbar instrumentation with a single solid rod in adolescent idiopathic scoliosis and maintained good correction at three levels without major complications, infections, or revision surgeries and with satisfactory final function and clinical quality [31]. Kelly et al. also published good correction results, with a 64% correction of the primary curve. There was minimal curve change within the follow-up time (16.97 years) [32]. Sudo et al. favoured short anterior instrumentation with dual-rod systems for curve correction to preserve as many caudal vertebral planes as possible. They showed a mean correction of 74% at the final follow-up 21.6 years after short fusion. Their group comprised 13 patients with the lowest instrumented vertebra at the lower end vertebra and 17 patients with LIV one level proximal to the end vertebra. However, coronal and sagittal balance, thoracic kyphosis, lumbar lordosis, and clinical outcomes evaluated by the Scoliosis Research Society-22 (SRS-22) questionnaire scores were similar in the two groups [33]. Rioullon et al. showed good coronal correction with an anterior screw plate system that was implanted by a thoraco-phreno-lumbotomy for different types of AIS. This surgical approach, however, is a highly invasive procedure, and plate osteosynthesis itself is no longer the standard for the management of AIS. In addition, correction in the sagittal plane was not sufficient using their procedure, and a markedly negative sagittal vertical axis (SVA) remained. Interestingly, they found a translation of the SVA from postoperatively to their last follow-up at a mean of 21 years. Furthermore, a correlation between the SRS-30 scores and the patient's pelvic tilt and SVA translation was demonstrated, which led the authors to believe that an anterior SVA translation over time may be associated with a better functional outcome [34].

In contrast, Darnis et al. reported better corrections following posterior thoracolumbar/lumbar fusion (between 72% and 79%) compared to the correction after anterior instrumentation and fusion in a cohort of 109 patients after a maximum follow-up of 20 years [35]. Good correction maintenance was achieved in 43 patients after a minimum follow-up of 15 years. The mean correction was 70% after the last follow-up visit. Furthermore, correction of the main thoracic curve led to a spontaneous lumbar curve correction in 57% of patients [36].

In summary, based on long-term observations, sufficient correction in the coronal plane is possible through anterior treatment. However, these studies do not provide clear statements about the postoperative positions in the sagittal profile and, therefore, do not correlate this factor with the clinical outcome. Furthermore, the extent of scoliosis correction does not directly correlate with the clinical outcome [37, 38].

From a biomechanical point of view, the length of the anterior/posterior fusion as well as the number of lumbar spine segments involved in the fusion could also be a factor in treatment outcome. In this context, Lavelle et al. found no significant difference in the long-term outcome (after a mean of 20 years in 22 patients treated using the CD technique) related to the length of spinal fusion. However, the follow-up in this retrospective study was solely carried out using questionnaires (SRS-22; SF-36; ODI; VAS), and radiological examinations were not part of the study [6]. However, most studies have shown that fusion to the lower lumbar spine worsens the outcome of AIS patients and that selective thoracic fusion should be performed if it is possible [14, 39–41]. Various approaches have been developed for the surgical treatment of adolescent idiopathic scoliosis (AIS), each with its advantages and drawbacks. For instance, the posterior approach is highly invasive but allows for significant corrections, whereas the anterior approach is less invasive but typically limited to the lower thoracic and lumbar spine. Furthermore, anterior approaches can provide a sufficient correction in the coronal plane, but in the case of global sagittal imbalance, anterior approaches could lead to insufficient restoration of the sagittal alignment.

### Proximal, distal junctional kyphosis and adjacent segment degeneration

The risk of adjacent segment degeneration (ASD) is a well-known problem in the surgical treatment of difficult spinal diseases. In such conditions, an increased load on the adjacent segments above and below the instrumented spinal levels seems to be caused by the iatrogenic-modified lever arm [42, 43]. Changes in the adjacent segments involve facet joint arthrosis, segmental instability, spinal stenosis, accelerated disc degeneration, and the development of spondylolysis in rare cases [44]. Besides the above-mentioned radiological findings for ASD, adjacent segment disease (ASDi) presents with pain after a symptom-free interval [45]. To reduce the occurrence of adjacent segment failure, several parameters have to be considered as potential risk factors related to the onset of ASD. Therefore, detailed evaluation of parameters such as stiffness of combined posterior/anterior instrumentation and fusion, consideration of lumbar lordosis, sagittal balance, knowledge of spinopelvic parameters, and the lowest instrumented vertebra belong to the surgical preparation [46]. Luk et al. reported hypermobility within the caudally adjacent levels after posterior fusion as an additional risk factor for ASDi in a 13-year follow-up in 62 patients. This might be casual for early disc degeneration within the hypermobile segments [47]. The role of the lowest instrumented vertebra in treating thoracic idiopathic scoliosis remains controversial in the literature. Beyond the primary objectives of maintaining sagittal and coronal balance and preserving motion segments, choosing the lowest instrumented vertebra seems to directly influence the onset of degeneration in adjacent segments, particularly in the distal junctional region [48]. In this context, DJK was significantly more likely to occur in the posterior group compared to the anterior fusion of thoracic curves (*p* < 0.001) [49]. Hamzaoglu et al. included 43 patients with a maximum radiological follow-up of 18 years [36]. The lowest posterior instrumented vertebra was Th11 in 4, Th12 in 25 and L1 in 14 patients. During the last follow-up visit, they described non-significant degenerative changes compared to the control group, especially at the L4/L5 and L3/4 levels. Kelly et al. showed similar results in patients following single anterior instrumentation. They described increased disc angulation and significant degeneration below the lowest instrumented vertebra after the fusion of the thoracolumbar curves but without clinical implications related to HQRoL [32]. Fischer et al. focused on the optimal lowest instrumented vertebra after posterior instrumentation and concluded that DJK is more likely to occur if: 1. the lowest instrumented vertebra is three or more levels proximal to the neutral vertebra, 2. the centre sacral vertical line is outside the lowest instrumented vertebra, and 3. ether Risser stage 0 or open triradiate cartilage or 4. a lumbar C modifier is present [50]. Takahashi et al. confirmed these results. They recommend stopping when the lowest instrumented vertebra is at, or at least one level distal to the stable vertebra. This distal lowest instrumented vertebra did not result in an increased rate of truncal imbalance or adjacent segment degeneration in their study [51].

Degenerative disc disease is also considered to be a late complication after AIS surgery [52, 53]. However, Chiu et al. found no difference in the degeneration of the remaining unfused lumbar intervertebral disc with the selection of the lowest instrumented vertebra. Still, they postulated that patients with fusion to L4 or lower had more significant back pain after a mean follow-up of 17.7 years [54]. After the correction of the hypokyphosis and the de-rotation of the apex, the most caudal fused vertebra must be horizontalised and translated into the stable zone [9].

The question of how many motion segments can remain unfused for the optimal correction of scoliosis, hypokyphosis, and de-rotation of the vertebral index curve cannot be answered definitively, because a reference value is lacking, and the guidelines for the optimal length of fusion vary in the literature.

The onset of proximal junctional kyphosis (PJK) is a common complication after surgical correction of the curves, leading to pain, adjacent deformity and even revision surgery. The main risk factors are larger preoperative kyphosis angle, greater immediate postoperative decrease in thoracic kyphosis angle, and male gender [48]. Yagi et al. observed 157 patients retrospectively and found the onset of PJK in 20% of their patients. Fusion to the sacrum and posterior fusion with segmental instrumentation have been identified as risk factors, although PJK can be minimised by postoperative normalisation of the global sagittal alignment [55].

Causes of ASDi are complex, and there are often multiple reasons for failure at the end of the construct. In addition to the different curve types, patients’ habitus, fusion length, the surgical approach and other factors can be causal. Based on the currently available long-term studies on AIS, no clear statements can be made about the extent to which a progressive ASDi in long-term follow-up is clinically relevant. The available studies do not offer any clear conclusions regarding the relation between radiological and clinical outcomes given by their inhomogeneity.

### Clinical outcomes

Several studies have focused on clinical outcomes after various types of surgical treatments for adolescent idiopathic scoliosis. The main health-related questionnaires used to assess quality of life were the Oswestry Disability Index (ODI), the Scoliosis Research Society (SRS), the visual analogue scale (VAS), and the short form-12 and -36 (SF-12/SF-36).

Rioullon et al. presented the results of 34/35 patients after a follow-up of 21 years and showed a mean SRS-30 score of 3.65/5 and a mean ODI of 14.9%. Furthermore, they focused on the onset of pain after surgical treatment of AIS. They reported pain at the cephalad end of the construct in 21/35 cases, low back pain in 26 cases, nerve root pain in five and intercostal neuralgia in four cases. However, very few patients reported severe pain [34]. Yamada et al. found a total SRS-30 score of 4.1 in Lenke Typ 1 AIS patients with upper instrumented vertebra (UIV) translation of < 20 mm and 3.9 in patients with a UIV > 20 mm, respectively, at their 18-year follow-up [56]. Lavelle et al. reported a mean SRS-22 score of 4.15 in their long outcome investigation, improving the score in patients with longer follow-ups. The group also found a mean SF-36 result of 72.05, an ODI of 15.36 and VAS back pain of 2.5 after a 15–26-year follow-up [6]. Akazawa et al. compared SRS-22 and low back pain (Roland Morris Disability Questionnaire; RDQ) outcomes of surgically treated patients with AIS to non-idiopathic scoliosis and a healthy control group at a minimum follow-up of 21 years. The authors found no significant differences in function, self-image or pain between the scoliosis groups. The idiopathic and non-idiopathic scoliosis groups performed worse in function, self-image, and RDQ results than the healthy control group. However, the results of this study must be interpreted with caution, because the composition of the idiopathic scoliosis group was not described in detail, and there were no evaluable data regarding the radiographs from the follow-up investigation. Matters were further complicated, because the patients in this study were treated with surgical implants that differ from those used today [57]. Takayama et al. administered the SRS-22 and SF-36 questionnaires at a mean follow-up of 21.1 years in 32 patients. Eighteen patients had AIS; eight were treated using the CD technique, seven using Harrington rods, and three using anterior surgical procedures. No impairments in the QoL, particularly in the AIS group were reported. Patients treated by the CD technique showed the best SRS-22 scores, while patients treated with anterior procedures showed worse scores. However, neither the level of distal fusion, Cobb angle preoperatively or at the latest follow-up, nor degenerative changes in the subjacent segment had any effect on the incidence of low back pain in these studies, and only sagittal balance represented a risk factor for lower back pain [5, 38, 58].

### Pregnancy and family planning

Kino et al. focused on HrQoL following surgical posterior fusion of AIS in women. Although HrQoL scores (SF-36) were lower than those of the healthy control group, the effects of posterior spinal fusion on women's social life and reproductive statuses were minimal [59]. Since the incidence of AIS in young females is approx. 8–10 times higher than in men, questions over their ability to give birth normally and possible complications of pregnancy arise [1, 2]. Takayama et al. found that 17 of 18 AIS patients in their study were employed at the final follow-up. In this cohort, 12 of 18 patients were married, and 9 of 15 delivered a mean of 1.78 children with a C-section rate of 18.75% [58]. Other studies have confirmed these results. Rioullon et al. reported that 21 out of 29 AIS females had a pregnancy during follow-up [34]. Akazawa et al. published similar rates of 1.7 deliveries per patient in their idiopathic scoliosis group at a minimum of 21 year follow-up and a marriage rate of 69.6% [57]. Swany et al. showed that the rate of C-sections in AIS patients was significantly higher compared to the national C-section rate. Furthermore, no differences in the C-section rate and the length of instrumentations were found. In contrast, some studies suggest decreased lumbar lordosis may be associated with increased C-section rates [40, 60]. The reason for this could be that a decreased lumbar lordosis, which refers to a reduced inward curvature of the lower spine, can affect the alignment and shape of the pelvis. This change can potentially narrow the birth canal, making vaginal delivery more difficult and increasing the likelihood of complications that necessitate a C-section. Other reasons could be impairment of the pelvic floor muscles. In this context, lumbar lordosis plays a role in the biomechanics and function of the pelvic floor muscles. Reduced lordosis might lead to suboptimal functioning of these muscles, which are crucial for supporting the uterus and aiding in the birthing process. Furthermore, decreased lordosis may alter the distribution of forces and pressures during labour, potentially leading to labour dystocia, a common reason for C-sections.

### Prospects

The development of idiopathic scoliosis will remain a problem in the future and will impact patients until the end of their lives. However, new surgical techniques and surgical materials, such as screw designs, reduction tools, navigation tools and robot-assisted procedures, are currently on the rise [61, 62]. These “future tools” will improve the surgical therapy of scoliosis patients within the next few years. Furthermore, better imaging will allow for even better preoperative and intraoperative planning in the future [63]. Even better reductions and shorter fusion distances will be possible to ensure long-term therapeutic success.

## Conclusions

Various fusion techniques are used for AIS and compete as the best surgical treatment. In rare cases, combined anterior and posterior techniques can increase the possibility of reduction. ASD remains a problem in fusion surgery, but given the weak long-term data, its clinical relevance in AIS patients cannot be scientifically proven. There is no evidence that AIS affects pregnancy ability, although reduced lumbar lordosis appears to be associated with higher C-section rates. In summary, it is difficult to compare the long-term data from the past with the surgical results from today due to the constantly improved treatment options. Still, our study shows that surgically-treated AIS patients' long-term outcome already seems good. Further studies, particularly prospective randomised control trials, are necessary to monitor the effect of treatment strategies.

## Data Availability

The datasets generated during and/or analysed during the current study are available throughout the manuscript.
